# Cluster-Based Statistics for Brain Connectivity in Correlation with Behavioral Measures

**DOI:** 10.1371/journal.pone.0072332

**Published:** 2013-08-19

**Authors:** Cheol E. Han, Sang Wook Yoo, Sang Won Seo, Duk L. Na, Joon-Kyung Seong

**Affiliations:** 1 Department of Biomedical Engineering, Korea University, Seoul, Republic of Korea; 2 Department of Computer Science, Korea Advanced Institute of Science and Technology (KAIST), Daejeon, Republic of Korea; 3 Department of Neurology, Sungkyunkwan University of Medicine, Seoul, Republic of Korea; 4 Department of Neurology, Samsung Medical Center, Seoul, Republic of Korea; Newcastle University, United Kingdom

## Abstract

Graph theoretical approaches have successfully revealed abnormality in brain connectivity, in particular, for contrasting patients from healthy controls. Besides the group comparison analysis, a correlational study is also challenging. In studies with patients, for example, finding brain connections that indeed deepen specific symptoms is interesting. The correlational study is also beneficial since it does not require controls, which are often difficult to find, especially for old-age patients with cognitive impairment where controls could also have cognitive deficits due to normal ageing. However, one of the major difficulties in such correlational studies is too conservative multiple comparison correction. In this paper, we propose a novel method for identifying brain connections that are correlated with a specific cognitive behavior by employing cluster-based statistics, which is less conservative than other methods, such as Bonferroni correction, false discovery rate procedure, and extreme statistics. Our method is based on the insight that multiple brain connections, rather than a single connection, are responsible for abnormal behaviors. Given brain connectivity data, we first compute a partial correlation coefficient between every edge and the behavioral measure. Then we group together neighboring connections with strong correlation into clusters and calculate their maximum sizes. This procedure is repeated for randomly permuted assignments of behavioral measures. Significance levels of the identified sub-networks are estimated from the null distribution of the cluster sizes. This method is independent of network construction methods: either structural or functional network can be used in association with any behavioral measures. We further demonstrated the efficacy of our method using patients with subcortical vascular cognitive impairment. We identified sub-networks that are correlated with the disease severity by exploiting diffusion tensor imaging techniques. The identified sub-networks were consistent with the previous clinical findings having valid significance level, while other methods did not assert any significant findings.

## Introduction

The recent trend of the human brain research is modeling the whole brain as a network, which consists of nodes, corresponding to brain regions, and edges, representing relationship between any pair of two brain regions. Graph theoretical approaches revealed novel characteristics of brain organization, including small-world topology [1]–[4], scale-free characteristics [4]–[6], global integration [1], [7]–[9], modular structure [4], [10], [11], and hierarchical organization [12]–[14] of the brain networks. For this purpose, various network topological measures have been suggested, which capture global characteristics of the whole brain. Those measures have been successfully exploited in contrasting patients with some diseases from healthy subjects. For example, reduced network efficiency of the structural connectivity was observed in patients with schizophrenia [15], [16], and a decrease in global clustering of the functional connectivity was reported in patients with Alzheimer’s disease [17]. Focusing on the global organization of the brain network, the graph theoretical approaches have been trying to understand how the complex interaction between brain regions would moderate cognitive functions [18].

Localization of abnormal brain connectivity is another primary research goal of the brain network analysis with regard to deterioration of a specific brain function. A large number of the Tract Based Spatial Statistics (TBSS) studies in clinical populations showed wide interests in localized analysis of abnormal white matter brain connectivity [19]–[23]. Furthermore, the disconnection hypothesis [24]–[26] provides a motivation for such a connectivity comparison analysis, which states that abnormal interaction between brain regions causes disease symptoms. It was employed in the neuroimaging studies to explain abnormal functional connectivity in patients with schizophrenia [27], [28] and developmental dyslexia [29] in the late 1990s, and expanded to seeking abnormality of both structural and functional connectivity in patients with various diseases [15], [30]–[35]. A localized analysis of brain connectivity that is responsible for a specific brain function also helps to plan an effective neurosurgery. For example, brain-tumor resection surgeries can minimize aphasia due to unintended excessive removal of the articulate fasciculus [36], [37], which plays an important role in lingual functions [38], [39].

Besides the group comparison analysis of brain connectivity, the identification of specific brain connections that lead behavioral changes is also a challenging research problem. In studies with patients, it would be interesting to understand which connections indeed deepen specific symptoms, while for studies involving subjects with normal cognition, a specific set of brain connections can be associated with normal aging process. However, seeking for connectivity correlated with specific behavioral measures has been less studied compared to studies seeking for group differences in connectivity between healthy and clinical populations. One of the major difficulties of such correlational studies in connectivity is too conservative multiple comparison correction. If we conduct a correlational study between a single behavioral measures and a certain global characteristic of the brain network, computing a correlation coefficient and estimating its significance level is enough to assert a statistically meaningful relationship between them. For example, a significant correlation was observed between global network properties such as global efficiency and behavioral measures including severity scores in structural connectivity [15] and functional connectivity [40], or intelligence quotients (IQ) in structural connectivity [16], [41] and functional connectivity [42]. However, if we performed a correlational study for numbers of network connections (edges), multiple comparison correction over independent hypotheses is necessary to assert statistically valid local correlations. Since biomedical data generally have weak correlations, too conservative multiple comparison correction procedure may often invalidate most of the findings, leading a large number of false negatives. As an example, if the network consists of 90 nodes, the number of all the edges is 4005. The most conservative correction method, Bonferroni procedure [43], will reject null hypotheses only for the edges of which *p* values are below the α-level divided by 4005; the corrected α-level is around 0.00001 for a traditional 5% α-level, which is difficult to attain in the neuroimaging data. In this study, we propose a cluster-based method for multiple comparison correction in connectivity analysis with correlation coefficients, in order to report statistically valid results while avoiding excessively conservative correction.

Similar to the underlying insight of the cluster-based statistics in voxel-based morphometry (VBM) [44]–[48], it is also valid that abnormal sub-networks, clusters of multiple connected edges, are responsible for abnormal behaviors than a single edge disrupts normal behaviors [49]. On the line of this insight, the Network-Based Statistics (NBS) [16], [49], [50] and the Spatial Pairwise Clustering (SPC) [51] were proposed to detect a sub-network that is significantly different between groups, and have been used successfully in many clinical applications [16], [52]–[54]. The essence of the SPC and the NBS is estimating statistical significance levels for abnormal sub-networks as a whole based on permutation testing, instead of performing multiple comparison correction of edges individually using the false discovery rate (FDR) procedure [55], [56]. Since the cluster-based statistics was first introduced to neuroimaging fields in 1993 [47], its statistical validity has been studied for voxels [57] and networks [49] using either the random field theory or the permutation test [44]–[48], [57]–[60]. Our approach inherits the permutation-based cluster analysis.

Adopting the cluster-based approaches above, our method identifies sub-networks of the brain connectivity that are significantly correlated with behavioral measures by clustering network connections. Specifically, our method captures direct correlation with cognitive functions using a partial correlation coefficient for each edge. As a result of group analysis, the NBS provides implicit information on the correlation of brain connectivity with a combination of several cognitive functions that are generally deteriorated in a certain disease, rather than a specific cognitive function. We emphasize that as correlating each edge directly with a specific behavioral measures, our method does not require healthy populations to contrast, and can identify sub-networks that capture a specific change of cognitive functions in patients, not all of their symptoms. Finding normal controls is often difficult, especially for old-age patients with cognitive impairment where controls could also have cognitive deficits due to normal ageing. It is also worthy to note that correlation coefficients can easily resolve the normality issue of the connectivity data. Because the general linear model framework relies on the normality assumption of the data, its application to the connectivity data could be less valid. In order to resolve the problem, we employed the non-parametric Spearman correlation coefficients. Use of partial correlation coefficient is critical in order to reduce the effects of the other compounding factors in the analysis. For example, cognitive deficits in patients with cognitive impairment may be combined with normal ageing.

Finally, we validated the proposed method using the brain connectivity data for patients with subcortical vascular cognitive impairment (SVCI). SVCI is a type of cerebrovascular disease, which led lowered cognitive functions [61]. It is well-known that deterioration in white matter nearby subcortical regions deepens the disease symptom [61], and thus, the structural connectivity of white matter also decreased. We compared our method to other multiple comparison correction methods: Bonferroni correction [43], FDR procedure [55], [56], and extreme statistics [62]. Our method was sensitive enough to identify sub-networks that deepen the SVCI-related symptoms, while no other methods can provide statistically significant results. We believe that the proposed cluster-based statistics enables more powerful multiple comparison correction in the brain connectivity correlational analysis, which is also simple to use in clinical applications.

## Methods

### Overview

In this section, we present a cluster-based method for identifying sub-networks of brain connectivity that are correlated with behavioral measures. Similar to the NBS, our scheme aims to control the family-wise error rate (FWER) when mass-univariate testing is performed at every connection of the network. The proposed method takes as inputs a set of connectivity matrices for all subjects in a given group and their corresponding behavioral measures. Our scheme is independent of a specific method for constructing a connectivity matrix: both structural and functional connectivity can be used according to the desired purpose. Therefore, the main focus of this section is the identification method for sub-networks of the brain connectivity matrix.

**Figure 1:**
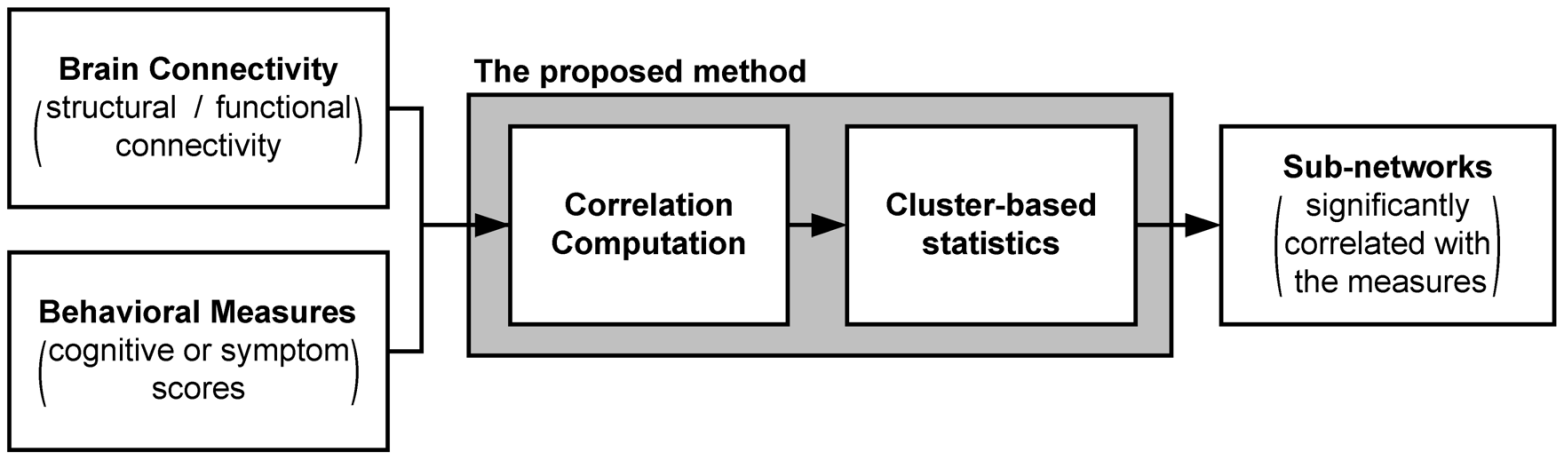
Overview of the proposed method. The method consists of two parts: correlation coefficient computation and multiple comparison correction with cluster-based statistics. In the former part, a partial correlation coefficient is calculated for each connection of the brain network with the behavioral measures. In this step, several compounding variables are taken as covariates in order to count their effects on the correlation coefficients. In the later part, we perform cluster-based correction for the multiple comparison of the correlation coefficients by adopting the supra-threshold cluster size test to our problem setting. In this approach, clusters are constructed by grouping together neighboring supra-threshold connections, and the *p*-values are estimated through permutation testing, forming a null distribution of the maximum cluster extent. The output of this step is a set of sub-networks consisting of neighboring connections that are significantly correlated with the behavioral measures.

As shown in Figure 1, the proposed method consists of two parts: correlation coefficient computation and cluster-based multiple comparison correction. In the former part, a partial correlation coefficient is calculated for each connection of the brain network with the behavioral measures. In this step, several compounding variables are taken as covariates in order to count their effects on the correlation coefficients. In the latter part, we perform cluster-based correction for the multiple comparison correction of the correlation coefficients by adopting the supra-threshold cluster size test [44], [46]. In this approach, clusters are constructed by grouping together neighboring supra-threshold connections, and the *p*-values are estimated through permutation testing, forming a null distribution of the maximum cluster extent. The output of this step is a set of sub-networks consisting of neighboring connections that are significantly correlated with the behavioral measures and the corresponding (corrected) *p*-values. We implemented the proposed method using Matlab 8.0 (32bit version, R2012b, Mathworks, Natick, USA), and uploaded both the codes and the test data set at http://bia.korea.ac.kr/people/~cheolhan/software/.

### Computing correlation coefficients

In the first step of our method, we compute partial correlation coefficients between each edge and a behavioral measure of interest independently, by taking other behavioral measures as covariates. Either Pearson or Spearman correlation coefficients can be employed, though we employed Spearman rank correlation coefficient since distribution of the brain connectivity data is unknown and often does not satisfy the normality condition. Based on a permutation testing, we estimate the significance level of the correlation coefficient by randomizing the order of the behavioral measures [63]. Permutation testing has been popular in neuroimaging and brain network analysis due to unknown nature of dataset distribution, which frequently violates normality assumption (See Ludbrook and Dudley [64] for more discussion).

**Figure 2:**
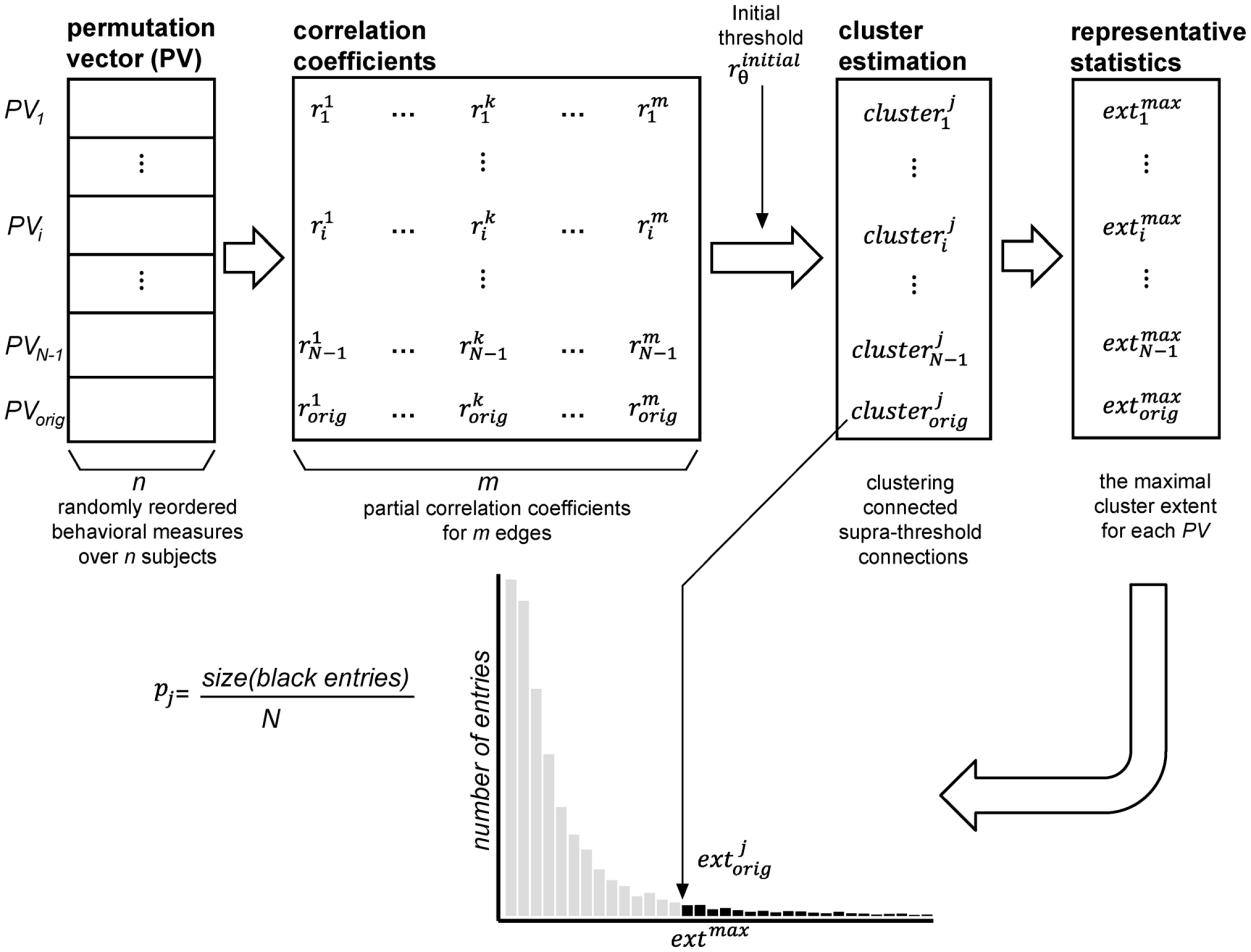
Cluster-based statistics of correlation coefficients for multiple comparison correction. The first step generates *N* permutation vectors by randomly reordering behavioral scores (the upper-left corner). Suppose there are *n* subjects in the given group. Then, the *i*th permutation vector *PV*_i_, *i* = 1,2,…,*N*, has *n* elements, the ordering of *n* subjects’ behavioral scores. Note that the last permutation vector *PV*_N_ is constructed using the original ordering of the behavioral scores as usual in any permutation testing. We compute a partial correlation coefficient between an edge and the behavioral scores for every permutation vector. We repeat this procedure for every edge, resulting 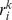, *i* = 1,2,…,*N* and *k* = 1,2,…,*m*, where m is the total number of edges (the upper middle). In the second step, we extract sets of network edges of which correlation coefficient is beyond the initial threshold 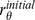 to form supra-threshold clusters. Denoted by 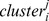 the resulting cluster is corresponding to the *j*th cluster of the *i*th permutation vector *PV*_i_, *i* = 1,2,…,*N* and *j* = 1,2,…*c_i_*, where *c_i_* is the number of identified clusters for *PV*_i_. For a positive initial threshold, edges whose correlations were larger than it will form clusters, while for a negative threshold edges whose correlation is smaller than it will do. We employ the maximum cluster extent for the null permutation distribution by counting the number of edges in the largest connected sub-network of each permutation. 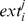 represents the number of edges in 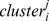, and 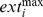
represents the maximum cluster extent for *PV*_i_ (the upper-right corner)_._ This representative statistic forms a null permutation distribution, which is shown as the histogram (the bottom). Finally, we estimate the significance level over the null distribution by computing the proportion of the number of entries whose maximal cluster extents are larger than the size of each identified sub-network, 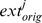, (black entries in the histogram) to the number of entries, *N*.

As shown in Figure 2, we first generate *N* permutation vectors by randomly reordering the behavioral measures (Figure 2, the upper-left corner). Suppose there are *n* subjects in the given group. Then, the *i*th permutation vector *PV*_i_, *i* = 1,2,…,*N*, is the *i*th random ordering of the *n* subjects’ behavioral measures. Note that the last permutation vector *PV*_N_ is constructed using the original ordering of the behavioral measures as usual in any permutation testing. Then, we compute a partial correlation coefficient between an edge and the behavioral scores for every permutation vector. We repeat this procedure for every edge, resulting 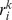, *i* = 1,2,…,*N* and *k* = 1,2,…,*m*, where *m* is the total number of edges. We note that because our main interest is observing the effect of the specific behavioral measures, we only reordered the measure of interest, while the covariates’ orders were kept.

For each edge, this repeated computation forms a null distribution of its correlation coefficient. The null distribution shows how the order of the behavioral measure affects its correlation coefficient. When the measure in the original order is highly correlated with the edge weight, the magnitude of the edge’s correlation coefficient is larger than the correlation coefficients generated using the other random orders. Thus, its significance level is estimated as the proportion of entries whose correlation coefficients are greater than the correlation coefficient of the original order to the number of total entries, *N*. This *p*-value highly correlates to the traditional *p*-value approximated by transforming the correlation coefficient to *t*-statistics [65]. In our experiment, for example, Spearman correlation coefficient between the permutation-based p-value and the *t*-statistics-based one was larger than 0.99 (see the Clinical Application section for details). The resulting correlation coefficients for every random ordering of the behavioral measures will be used in the second step of our method (see the next section).

### Cluster-based statistics

The second step of our method performs cluster-based multiple comparison correction for correlation coefficients computed in the previous step for all network edges. It is common to perform thousands or millions of the same statistical tests in neuroimaging research; in our case, we tested correlation with a behavioral measure for 4005 edges. To avoid accumulation of Type-I error due to multiple comparisons, the significance level of each test could be corrected through either Bonferroni procedure [43] or FDR procedure [55], [56]. Otherwise, one can employ permutation based methods for the multiple comparison correction: extreme statistics [62] and cluster-based statistics [44]. Those two methods generalize the randomization procedure to a family of tests, forming a permutation distribution of a certain representative statistic, which results in less conservative correction than the others. Our method employed the cluster-based statistics among them. Specifically, we use the maximal size of each cluster as a representative statistic, where a cluster consists of connected supra-threshold edges for an arbitrary initial threshold [44], [49]. Then a significance level of each cluster is estimated by its size on the null distribution, capturing the cluster occurrence probability with the score of the original ordering in our case. This cluster-based statistics has been successful in neuroimaging with regard to voxels [20], [44], [46], [57], [58], [60], vertices of cortical surfaces [66]–[68], and network edges [16], [49], [52]–[54] in group comparison.

Our method exploits the maximum size of the cluster as a representative statistic (Figure 2). Specifically, we first extract sets of network edges of which correlation coefficient is beyond the initial threshold 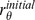 to form supra-threshold clusters using the breadth first search algorithm of the Matlab Boost Graph Library [69]. Denoted by 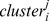 in Figure 2, the resulting cluster is corresponding to the *j*th cluster of the *i*th permutation vector *PV*_i_, *i* = 1,2,…,*N* and *j* = 1,2,…*c_i_*, where *c_i_* is the number of identified clusters for *PV*_i_. For a positive initial threshold, edges of which correlations are larger than the threshold will form clusters, while for a negative threshold edges of which correlations are smaller than the threshold will do. Though the selection of an initial threshold may affect the identified networks, the significance level calculated by this procedure is always valid [20]. We employ the maximum cluster extent for the null permutation distribution by counting the number of edges in the largest connected sub-network of each permutation. In Figure 2 (the upper-right corner), 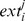 represents the number of edges in 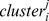, and 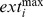 represents the maximum cluster extent for *PV*_i._ This representative statistics form a null permutation distribution, which is shown as the histogram in the bottom of Figure 2. Finally, we estimate the significance level over the null distribution by computing the proportion of the number of entries whose maximal cluster extents are larger than the size of each identified sub-network, 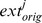, to the number of entries, *N*. Precisely, *p_j_*, the significance level of 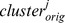 is given by
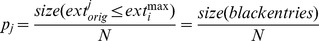



where *size*(•) counts the number of entries.

In summary, given brain networks and behavioral measures, the proposed cluster-based method identifies a set of sub-networks consisting of neighboring brain connections that are strongly correlated with the behavioral measures, and also provides the corresponding *p*-values (corrected) for each sub-network.

### Comparison to the other multiple comparison correction methods

In order to show the efficacy of our method, we compare it with other multiple comparison methods: Bonferroni correction [43], false discovery rate (FDR) procedure [55], [56], and extreme statistics [44], [46], [60], [62]. The Bonferroni correction method uses a corrected α-level, which is simply divided by the number of hypotheses. If we test for *m* edges, the corrected α-level will be α/*m*: it rejects null hypotheses only for the edges of which *p*-values are below the corrected α-level. The FDR procedure controls the ratio of false positives to all findings (positives). Specifically, we sort the *p*-values in an ascending order and find the largest *p*-value which is not greater than α*k*/*m* where *k* is the order of the *p*-value in the sorted list. Then, any edge of which *p*-value is not greater than the largest *p*-value is asserted as a significant finding.

In order to perform the permutation-based extreme statistics, the greatest magnitude of the correlation coefficients were used as a representative statistic for each permutation vector [62]. To identify positively correlated connections, we collected the maximum of correlation coefficients for each permutation vector, while for negatively correlated connections, we used their minimum values. More precisely, in figure 2, we used 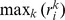 or 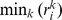 as a representative statistic for *PV*_i_, *i* = 1,2,…,*N* and *k* = 1,2,…,*m*, instead of 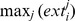. Then, we selected a single global threshold, 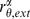 to control the experiment-wise α-level [46]. A significance level of each supra-threshold connection was estimated by locating observed correlation coefficients on the null distribution.

Because Bonferroni correction and FDR procedure are performed for *p*-values which do not carry the sign information of the edges’ correlation coefficients, comparison of those methods with either our method or extreme statistics may be unfair, which only use one-side of the correlation coefficients. Thus, we performed Bonferroni correction and FDR procedure for edges with negative and positive correlations separately. We note that this is already less conservative than using all edges of which correlation coefficients are real-valued by reducing the number of edges to around half.

## Clinical Application

### Objectives

In this experiment, we aim to validate the efficacy of the proposed method using clinical data of patients with subcortical vascular cognitive impairment (SVCI). Specifically, we constructed a structural brain connectivity matrix for each patient by employing the diffusion tensor imaging technique, and we tested if each edge weight (strength) is correlated with severity of the disease. As a severity measure, we used the CDR-SOB score for each patient. The expected output of our method is a set of sub-networks consisting of neighboring connections that are significantly correlated with the disease severity. We further compared our method with Bonferroni correction, FDR procedure, and extreme statistics.

### Subject recruitment and MR image acquisition

We recruited 36 patients with subcortical vascular mild cognitive impairment (svMCI) and 41 patients with subcortical vascular dementia (SVaD). All subjects had been clinically diagnosed at Samsung Medical Center between October 2007 and August 2010. Patients with SVaD met the diagnostic criteria for vascular dementia as determined by the Diagnostic and Statistical Manual of Mental Disorders–Fourth Edition (DSM-IV). All SVaD patients exhibited signficant ischemia as determined by MRI scans, defined as a cap or band ≥ 10 mm as well as a deep white matter lesion ≥ 25 mm (a modification of the Fazekas ischemia criteria [70]). The 41 SVaD patients have been previously described regarding clinical characteristics and [^11^C] PiB-PET findings [71]. Patients with svMCI were diagnosed using the Petersen criteria [72] with the inclusion of the following modifications: 1) subjective cognitive complaints by the patient or his/her caregiver; 2) normal Activity of Daily Living 3) objective memory decline assessment below the 16^th^ percentile on neuropsychological tests, 4) absence of dementia; and 5) presence of a subcortical vascular feature defined as both a focal neurological symptom/sign and significant ischemia on MRI, as for SVaD. We confirmed that no patient had territory infarctions or high signal abnormalities on the MRI due to radiation injury, multiple sclerosis, vasculitis or leukodystrophy. We included only patients with pure subcortical vascular cognitive impairment, by excluding patients who showed positive Pittsburgh compound-B (PiB) in PET scans [73], where patients were considered PiB-positive if their global PiB retention ratio was over 1.5 from the mean of the normal controls [71].

The study was approved by the Institutional Review Board of the Samsung Medical Center. We obtained written informed consent from all the participants. Structured written consent procedures were used by research staff when approaching participants with cognitive impairment. The assent of ‘‘next of kin’’ was required for participation of people with cognitive impairment who were unable to provide informed consent.

Severity of all patients was evaluated with a widely-used clinical dementia rating sum of boxes (CDR-SOB) score. It measures cognitive functions as a sum of six categories: memory, orientation, judgment and problem solving, involvement in community affairs, involvement at home and in hobbies, and personal care with the scale of 0, 0.5, 1, 2 and 3 for each item [74]–[77]. CDR-SOB is well matched with the global staging score, and varies enough to be treated as a quantitative score for a statistical test [76], [78]. It is also beneficial for evaluating mild cognitive impairment [79] due to its nature of higher resolution than the global staging score. We also measured the mini mental state examination for Korean (K-MMSE) [80], [81].

T1 and diffusion weighted images (DWI) were acquired from all 77 subjects at Samsung Medical Center using the same 3.0 T MRI scanner (Philips 3.0T Achieva). T1 weighted MRI data was recorded using the following imaging parameters: 1 mm sagittal slice thickness, over-contiguous slices with 50% overlap; no gap; repetition time (TR) of 9.9 ms; echo time (TE) of 4.6 ms; flip angle of 8°; and matrix size of 240×240 pixels, reconstructed to 480×480 over a 240 mm field of view. In the whole-brain diffusion weighted MRI examination, sets of axial diffusion-weighted single-shot echo-planar images were collected with the following parameters: 128×128 acquisition matrix, 1.72×1.72×2 mm^3^ voxels; reconstructed to 1.72×1.72×2 mm^3^; 70 axial slices; 220×220 mm^2^ field of view; TE 60 ms, TR 7383 ms; flip angle 90°; slice gap 0 mm; b-factor of 600 s/mm^2^.With the baseline image without diffusion weighting (the reference volume), diffusion-weighted images were acquired from 45 different directions. All axial sections were acquired parallel to the anterior commissure-posterior commissure line.

### Image preprocessing and network construction

We used the automated anatomical labeling (AAL) template [82], which contains 78 cortical regions and 12 sub-cortical structures. For correspondence between the AAL template and every subject’s diffusion weighted image, we nonlinearly registered the AAL template to their diffusion weighted images separately. For each subject, we first linearly registered the T1-weighted image to the reference volume of the diffusion image using the FSL Linear Registration Tool (FLIRT) [83], [84] and nonlinearly to the ICBM152 T1 template in the MNI space where the AAL template are defined using the FSL Nonlinear Registration tool (FNIRT) [85], [86]. An expert neuroanatomist (Dr. Sang Won Seo, one of the coauthors of this paper) visually validated this registration procedure. We mapped the AAL atlas to individuals’ diffusion spaces using the inverse of the nonlinear transformation from T1 spaces to the MNI space, and the linear transformation from T1 to diffusion spaces, with the nearest neighbor interpolation method to preserve the discrete labels of the AAL atlas. For diffusion weighted images, we corrected the eddy current distortions and the head motions by registering volumes with non-collinear diffusion directions to the reference image using FSL.

In order to quantify edge weights (strengths), we employed a deterministic tractography algorithm [87] implemented on the DTI-studio [88]. We initiated fiber tracking from voxels whose FA values are greater than 0.2, with following stopping criteria: 45 degree angle threshold and 0.2 FA threshold [89]. After finishing the whole-brain tractography, we counted the number of streamlines between any pair of regions defined by the AAL atlas, resulting in a 90×90 connectivity matrix for each subject. We discarded too weak edges whose number of streamline is below 3 to reduce artifact of tracking [90].

### Network Analysis

We performed permutation testing with 10000 permutations in order to identify connections that are correlated with the disease severity. Specifically, we computed a Spearman partial correlation coefficient between each edge weight and the severity score, CDR-SOB, by taking age and gender as covariates. The cognitive deficits of patients could be the combination of disease-related symptoms and cognitive changes due to normal ageing. The total number of streamlines could be affected by the gender. Thus the effects of age and gender need to be corrected by taking them as covariates. In this experiment, we observed that the CDR-SOB scores were not normally distributed (Lilliefors' test for normality [91], *p*<0.01). We therefore employed Spearman correlation for computing correlation coefficients.

In our cluster-based method, we systematically searched the initial threshold, 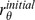, within the range between −0.5 and 0.5 with 0.01 step-size. The results were then reported with empirically selected thresholds, which provided stable clusters over multiple runs with different permutation vectors and disturbance of the initial thresholds. We used both 5% and 10% α-level for Bonferroni correction and the extreme statistics, and *q*-values of 0.05 and 0.1 for the FDR procedure to compare with our method.

For all tables and figures, we used abbreviated labels of the AAL90 atlas (Table S1). To denote the left and the right hemisphere, we attached L and R at the end of each abbreviated name. For figures, we used in-house Matlab codes, which show nodes of the AAL90 and significant edges over a transparent cortical mesh surface of the freesurfer’s average subject [92], [93]. All analysis and visualization were performed using Matlab 8.0 (32 bit version, R2012b, Mathworks, Natick, USA).

### Demographic result

We recruited 36 svMCI and 41 SVaD patients (Table 1). Because our primary concern is not comparing brain connectivity between two groups but finding network connections correlated with the severity scores in each group independently, two groups do not have to be age and gender matched. However, for comparison of the identified sub-networks from each group, we controlled age, gender, and education duration between two groups. Specifically, age (two-sample *t*-test, *t* = 0.88, *p* = 0.38), gender (Chi-square test, *χ^2^* = 1.94, *p* = 0.16) and education duration (Wilcoxon’s ranksum test, *z* = 0.19, *p* = 0.85) of two groups were not different each other. SVaD patients had significantly lower cognitive functionality (KMMSE, ranksum test, *z* = 5.30, *p*<0.0001), and significantly higher severity (CDR-SOB, ranksum test, *z* = –6.65, *p*<0.0001) than those with svMCI. We note that other measures except age were not normally distributed (Lilliefors' test for normality, [91]): education duration (svMCI, *p* = 0.0036; SVaD, *p* = 0.0116), KMMSE (svMCI, *p* = 0.0382; SVaD, *p* = 0.0174), and CDR-SOB (svMCI, *p* = 0.0092; SVaD, *p* = 0.0028). Thus, we used non-parametric Wilcoxon’s ranksum test for group analysis. We confirmed that CDR-SOB itself was not correlated with age (Spearman, svMCI, *r* = 0.25, *p* = 0.15; SVaD, *r* = 0.09, *p* = 0.58), and gender (Spearman, svMCI, *r* = 0.10, *p* = 0.57; SVaD, *r* = –0.02, *p* = 0.92).

**Table 1:** Demography of participants.

Group	svMCI	SVaD	Comparison
The number of subjects	36	41	
Age (years)	73.22±5.91	71.88±7.36	*t* = 0.88, *p* = 0.38
Gender ratio (F/M)	11/25	20/21	*χ^2^* = 1.94, *p* = 0.16
Education duration (years)	8.44±5.13	8.15±4.89	*z* = 0.19, *p* = 0.85
KMMSE	26.75±2.01	21.80± 4.33	*z* = 5.30, *p* <0.0001*
CDR SOB	1.21±0.71	5.93±3,71	*z* = –6.65, *p* <0.0001*

* : significant, We used a 2-sample *t*-test for group difference in age, Chi-Square test for gender ratio difference, and Wilcoxon’s ranksum test for the others.

### Clinical result: svMCI study

The number of edges negatively correlated with the disease severity score (CDR-SOB) is more than double of the number of edges positively correlated with the severity score (1356 edges were negatively correlated with the severity score, and 526 edges were positively correlated with the severity score). We employed Bonferroni correction (α = 0.05 and 0.1), FDR procedure (*q* = 0.05 and 0.1) and extreme statistics (5% and 10% α-level) for multiple comparison correction, and no connection was significant after the corrections. We employed the Bonferroni correction and the FDR procedure for 1356 edges negatively correlated with the severity score for fair comparison.

**Figure 3:**
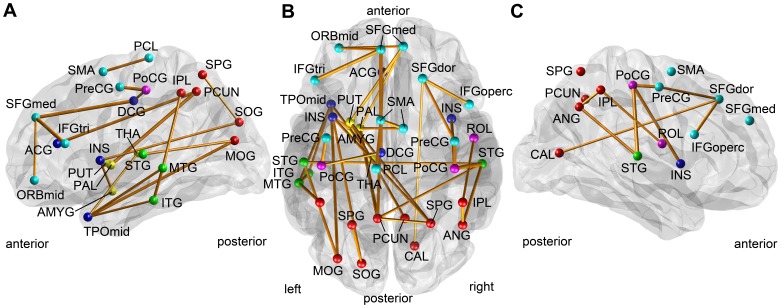
The identified sub-network correlated with the disease severity: subcortical vascular mild cognitive impairment. The figure shows in the lateral view of the left hemisphere (A), the transverse view of both hemispheres (B), and the lateral view of the right hemisphere (C). The identified connection was shown as an orange line, whose thickness represents the magnitude of its correlation coefficient between its edge weight and CDR-SOB. The identified node was shown with a colored sphere, whose color represents the lobe to which it belongs: frontal (cyan), limbic (blue), central (magenta), temporal (green), parietal and occipital (red).

On the contrary, the proposed cluster-based correction method provides a sub-network containing 38 connections (p = 0.0079, Figure 3, Table 2) with the initial threshold 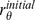 =  –0.39. We empirically selected the initial threshold, which provided stable clusters over multiple runs with different permutation vectors and small perturbation of the initial thresholds. Small changes in the initial threshold did not alter the result much. The results were significant when the initial threshold is located in between 0 and –0.408. No significant result was observed with a positive initial threshold.

**Table 2:** The identified sub-network correlated with the disease severity in patients with svMCI (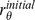 = –0.39, *p* = 0.0079).

Connections	*r*	*p*-value†	Connections	*r*	*p*-value†
PoCG.R - STG.R	–0.5517	0.0005	PreCG.R - SFGdor.R	–0.4276	0.0107
IFGtri.L - SFGmed.L	–0.5394	0.0012	PCUN.R - PUT.L	–0.4224	0.0131
PCUN.L - PAL.L ‡	–0.5119	0.0015	MTG.L - TPOmid.L	–0.4214	0.0126
SFGmed.L - DCG.L ‡	–0.5116	0.0023	INS.L - SPG.R ‡	–0.4203	0.0017
SMA.L - SMA.R	–0.5006	0.0025	DCG.L - PoCG.L	–0.4199	0.0014
PreCG.L - PoCG.L	–0.4983	0.0036	INS.L - AMYG.L	–0.4145	0.0109
PreCG.R - PoCG.R	–0.4894	0.0029	INS.L - SOG.L ‡	–0.4133	0.0162
INS.R - PoCG.R	–0.4763	0.0045	ORBmid.L - SFGmed.L	–0.4113	0.0123
SFGmed.R - PUT.L	–0.4740	0.0041	SPG.R - PUT.L	–0.4105	0.0123
ACG.L - PCUN.L ‡	–0.4700	0.0058	THA.L - TPOmid.L	–0.4102	0.0182
MOG.L - TPOmid.L ‡	–0.4671	0.0066	ROL.R - IPL.R	–0.4081	0.0144
MTG.L - ITG.L	–0.4660	0.0052	SMA.R - PAL.L	–0.4080	0.0186
SFGdor.R - IFGoperc.R	–0.4626	0.0058	PCUN.L - STG.R	–0.4039	0.0169
IPL.L - MTG.L	–0.4602	0.0069	SFGdor.R - CAL.R ‡	–0.4006	0.0152
ANG.R - STG.R	–0.4579	0.0065	SOG.L - SPG.L	–0.3990	0.0221
SMA.L - PCL.L	–0.4488	0.0093	STG.L - STG.R	–0.3987	0.0167
IPL.R - ANG.R	–0.4449	0.0097	ORBmid.L - SFGmed.R	–0.3986	0.0211
MOG.L - STG.L	–0.4446	0.0096	TPOmid.L - ITG.L	–0.3968	0.0190
SPG.R - PCUN.L	–0.4314	0.0134	SFGmed.R - PAL.L	–0.3956	0.0186

† : uncorrected, ‡: the anterior-to-posterior connectivity

### Clinical result: SVaD study

The number of edges negatively correlated with the disease severity score (CDR-SOB) is 50% more than the number of edges positively correlated with the severity score (954 edges were negatively correlated with the severity score, and 601 edges were positively correlated with the severity score). We employed Bonferroni correction (α = 0.05 and 0.1), FDR procedure (*q* = 0.05 and 0.1) and extreme statistics (5% and 10% α-level) for multiple comparison correction, and no connection was significant after the corrections. We employed Bonferroni correction and the FDR procedure for 1012 edges negatively correlated with the severity score for fair comparison.

**Figure 4:**
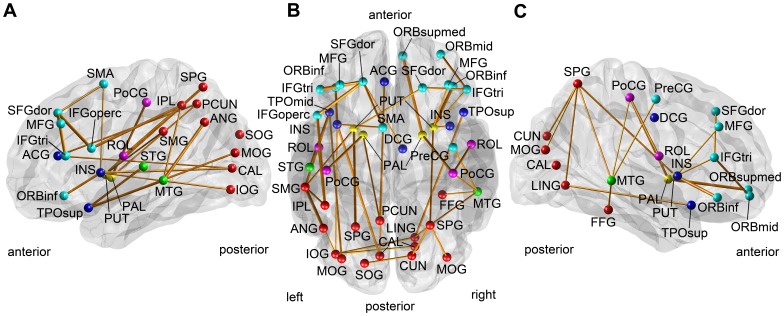
The identified sub-network correlated with the disease severity: subcortical vascular dementia. The figure shows in the lateral view of the left hemisphere (A), the transverse view of both hemispheres (B), and the lateral view of the right hemisphere (C). The identified connection was shown as an orange line, whose thickness represents the magnitude of its correlation coefficient between its edge weight and CDR-SOB. The identified node was shown with a colored sphere, whose color represents the lobe to which it belongs: frontal (cyan), limbic (blue), central (magenta), temporal (green), parietal and occipital (red).

On the contrary, our method identified a sub-network containing 45 connections (*p* = 0.0432, Figure 4, Table 3) with the initial threshold 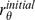 =  –0.32. We empirically selected the initial threshold, which provided stable clusters over multiple runs with different permutation vectors and small perturbation of the initial thresholds. Small changes in the initial threshold did not alter the result much. The results were significant when the initial threshold is in the range of –0.325 ≤ 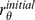
≤ –0.318 and –0.444 ≤ 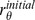
≤ –0.416 No significant result was observed with a positive threshold.

**Table 3:** The identified sub-network correlated with the disease severity in patients with SVaD (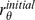 = –0.32, *p* = 0.0443).

Connections	*r*	*p*-value†	Connections	*r*	*p*-value†
SFGdor.L - IFGtri.L	–0.5040	0.0009	SPG.R - PUT.R ‡	–0.3555	0.0302
ORBinf.R - PUT.R‡	–0.4919	0.0022	SFGdor.R - IFGtri.R	–0.3540	0.0243
SFGdor.L - IFGoperc.L	–0.4901	0.0018	ACG.L - PCUN.L ‡	–0.3517	0.0281
ANG.L - MTG.L	–0.4787	0.0023	SFGdor.R - PUT.L	–0.3493	0.0263
ORBsupmed.R - PAL.R‡	–0.4492	0.0031	CUN.R - SPG.R	–0.3444	0.0353
IFGoperc.L - IPL.L ‡	–0.4438	0.0045	PreCG.R - MTG.R	–0.3396	0.0353
ROL.L - IPL.L	–0.4345	0.0050	PCUN.L - PAL.L ‡	–0.3382	0.0309
SPG.L - PUT.L ‡	–0.4268	0.0064	SMG.L - PUT.L	–0.3353	0.0328
TPOsup.L - MTG.L	–0.4239	0.0066	MOG.R - SPG.R	–0.3337	0.0383
PoCG.R - PAL.R	–0.4137	0.0098	CUN.R - SOG.L	–0.3335	0.0383
ORBinf.L - INS.L	–0.4098	0.0123	SPG.R - MTG.R	–0.3330	0.0386
INS.L - CAL.L ‡	–0.4085	0.0081	INS.R - PUT.R	–0.3327	0.0400
STG.L - TPOsup.L	–0.4071	0.0126	LING.R - SPG.R	–0.3322	0.0338
PoCG.L - PAL.L	–0.4064	0.0116	IOG.L - MTG.L	–0.3306	0.0388
ORBmid.R - IFGtri.R	–0.3818	0.0137	SMA.L - INS.L	–0.328	0.0387
ROL.R - PoCG.R	–0.3804	0.0174	IFGtri.R - PUT.R	–0.3279	0.0447
SFGdor.L - SMA.L	–0.3778	0.0189	FFG.R - MTG.R	–0.3266	0.041
LING.R - TPOsup.R ‡	–0.3753	0.0189	ACG.L - CAL.L ‡	–0.3253	0.0436
MOG.L - TPOsup.L ‡	–0.3744	0.0169	PoCG.R - MTG.R	–0.3228	0.046
CAL.R - IOG.L	–0.3673	0.0181	SFGdor.L - DCG.R	–0.321	0.0425
LING.R - IOG.L	–0.3659	0.0130	INS.L - IOG.L ‡	–0.3209	0.0444
SFGdor.L - MFG.L	–0.3642	0.0241	MFG.R - PUT.R	–0.3201	0.0442
IPL.L - MTG.L	–0.3576	0.0270			

† : uncorrected, ‡: the anterior-to-posterior connectivity.

### Reproducibility

In order to show reproducibility of our results, we repeated the same procedure for 10 randomly generated sub-sets of each group data. Specifically, we generated 20 age, gender, education duration, KMMSE, and CDR-SOB matched sets by randomly removing 10% of the subjects from each group. We then performed the proposed cluster-based statistics for the sub-sets separately. The results were compared to those of the original experiment: statistical significance and the identified sub-networks were reported for the comparison purpose. For the svMCI group, the reproducibility experiments showed that the results were statistically significant for all 20 random sets and the identified sub-networks contained more than 96% of the network connections extracted from the original experiment in average. For the SVaD group, 18 among 20 different random sets showed significant results, which contained more than 80 % of the network connections identified by the original experiment in average.

## Discussion

### Methodological issues

Our cluster-based analysis method identifies sub-networks of the brain connectivity associated with a specific cognitive function represented by a behavioral measure. A partial correlation coefficient is calculated for each edge with the behavioral score by taking other measures as covariates. The computation is repeated for randomly generated reordering of the behavioral scores. With a certain initial threshold, we then extract sub-networks of the brain connectivity by clustering supra-threshold connections. Finally, significance levels of the identified sub-networks are estimated using the permutation distribution of maximal cluster extents computed from each ordering of the behavioral measures.

**Figure 5:**
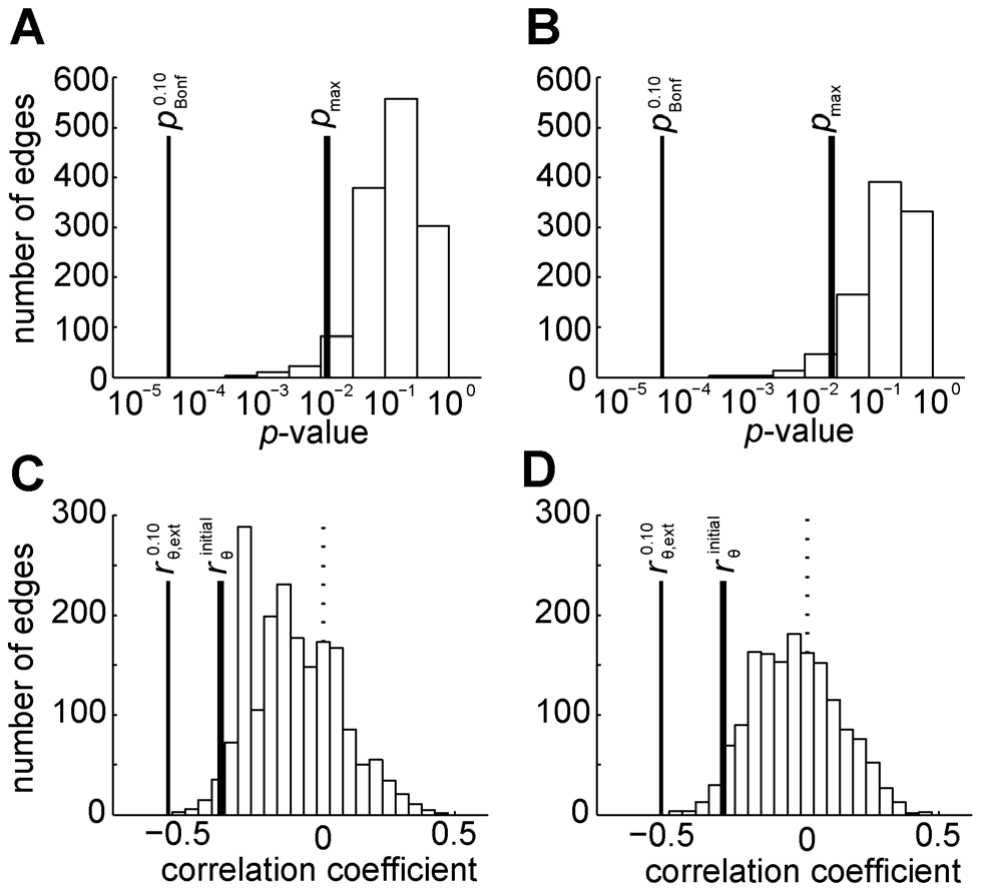
Comparison to the other multiple comparison correction methods. To compare with Bonferroni correction and FDR procedure, we drew histogram of *p*-values in log-scale whose correlation coefficients are negative, showing the thresholding *p*-value of Bonferroni correction with α = 0.10, 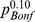 (thin solid vertical line), and the maximum of uncorrected *p*-values of network connections in the proposed cluster-based correction, *p*_max_ (thick solid vertical line), for patients with svMCI (A) and SVaD (B). We note that the thresholding *p*-values of the FDR procedure with q = 0.05 and 0.1 cannot be shown in log-scale, because they both are exactly zero, leading no significant findings. To compare with extreme statistics, we drew the histogram of raw correlation coefficients (Spearman, partial correlation adjusting age and gender), showing 10% threshold of the extreme statistics, 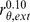 (thin solid vertical line), along with the initial threshold, 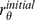
(thick solid vertical line), in patients with svMCI (C) and SVaD (D), where dotted vertical line indicates the zero correlation coefficient.

The proposed method was validated using clinical data involving patients with both svMCI and SVaD. In the experiment, we observed that our method was more sensitive in multiple comparison correction than other methods: Bonferroni correction, FDR procedure and extreme statistics. Bonferroni correction with α-level of both 0.05 and 0.10, and FDR procedure with *q* of both 0.05 and 0.10 did not assert any statistically significant findings. Their thresholding *p*-values were smaller than the largest *p*-value of the connections in the sub-network identified by our method (Figure 5 A&B, svMCI, *p* = 0.0221, SVaD, *p* = 0.0460). Though the extreme statistics is less conservative than Bonferroni correction and FDR procedure, it is still more conservative than the cluster-based correction (Figure 5 C&D). The magnitude of the selected initial threshold in the proposed method is smaller than that of the global threshold in the extreme statistics even with the α-level of 0.10 (svMCI, 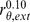 =  –0.59; SVaD, 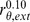 =  –0.56). This superior sensitivity of the cluster-based statistics is consistent with previous studies that compared cluster-based statistics with FDR [49], [50], [60]

Though our method shares the fundamental concept with that of the NBS, it is different from the NBS in three aspects. First, our method is for the correlational study not for group comparison. Thus, it captures direct correlation with cognitive functions without the healthy control group. Second, we can resolve the normality issue of the connectivity data by employing non-parametric Spearman correlation coefficients. Third, our method is simpler to use since we employ partial correlation coefficients for observing the effect of a certain cognitive measure controlling other effects. The current NBS toolbox can perform the regression study using the general linear model (GLM). The partial correlation coefficient counts the effects of the compounding factors, similar to the GLM. Since the GLM with the all factors only provides the overall goodness-of-fit with the all factors, to evaluate the sole effect of the factor of interest (in our clinical application, disease severity), we need to fit the GLM twice: with the factor of interest and without it.

Our method can be employed in various applications for identifying sub-networks correlated with a certain cognitive function. Task-specific approaches using functional MRI can also be employed for the same purpose. Specifically for the fMRI studies, when performing a certain cognitive task, synchronized activation of various brain regions may have a role in the cognitive function coherent with the task [94]. Hipp et al. localized the sub-networks correlated with the performance of the given task using the Spatial Pairwise Clustering (SPC), another form of cluster-based multiple comparison correction for network connections [50], [51]. However, our method is more beneficial than the method with task-specific functional MRI in two aspects. First, it does not require a special MR image recording, a task-specific functional MRI: instead, our method is independent of the brain network construction method. Second, the proposed method can be applicable to any behavioral measure which is not connected with a specific task, such as Intelligence Quotient (IQ), age, and disease severity.

Similar to the network based statistics (NBS) [49], the identified network connections in our method depend on the selection of an initial threshold, though the estimated significance level is always valid [20]. The NBS suggests testing for a threshold in a range of values. Accordingly, it is also recommended for our method to use a series of threshold values and find one with stable results. Unlike the NBS, however, selecting an initial threshold for correlation coefficient is more delicate than that for *t*-statistics. It is well-known that there exists a nonlinear mapping between correlation coefficients and *t*-statistics [65] (Figure S1). For small values of the correlation coefficient, the mapping is approximately linear. However, as the correlation coefficient increases, the corresponding *t*-value follows a super-linear function. Therefore, finer control is required for selecting an initial *r*-threshold as the threshold increases. Moreover, such a super-linear growth starts at earlier position as the number of subjects increases (Figure S1). We therefore recommend to tune the initial threshold more carefully at larger correlation coefficients and also for bigger group size. Under this circumstance, one may want to first convert the correlation coefficient to the corresponding *t*-value, and then control the initial threshold. However, this is not feasible in the case of employing the Spearman partial correlation coefficients since the mapping between the Spearman correlation coefficient and the *t*-statistics is not well-established.

It would be interesting to compare our method with the analysis of network motifs [95], [96]. A network motif is a small characteristic sub-network, which could play a role of building blocks of the complex networks. While both the motif analysis and the proposed method identify sub-networks without a control group, they are different in three aspects. First, a motif is a small set of brain connections (usually connecting 3–5 brain regions), but our method asserts bigger sub-networks in general. Second, implication of the identified connection is different. In the motif analysis, an identified edge implies existence of the connection. However, in our method, each edge in the identified sub-networks contains a correlation coefficient between its weight and a behavioral measure of interest, and therefore it implies a significance level for the correlational study. Third, our method requires a group of subjects, while the motif analysis can be performed for a single subject. Thus the motif analysis requires additional measures such as the occurrence frequency of each network motif for a statistical test.

We further emphasize that the purpose of our method is not for differentiating one group from the other. As directly correlating the network connections (edge weights) with the behavioral measures, we identified sub-networks that are responsible for the changes represented by the behavioral measures. We note that the clustering technique has also been used for other purposes: clustering brain regions to investigate modular organization of the brain networks [12], [97], clustering brain regions in a feature space to find uncommon network motifs [98], [99], clustering streamlines extracted from the DTI to identify the major fiber bundles [100], [101], and clustering subjects in a feature space to differentiate structural networks of one group from the other [102], [103].

### Clinical Interpretations

In the clinical application involving patients with svMCI and SVaD, we identified sub-networks of which connections are correlated with the disease severity. All the identified connections were negatively correlated with the severity score for both groups. The result indicates that the white matter connectivity is becoming more disrupted in the identified sub-networks as the disease becomes more severe. We observed that the identified sub-networks were reliable in statistical significance levels even with greater magnitude of the initial threshold. Furthermore, the results were also reproducible for both groups. We therefore infer that the identified sub-networks were core sets of the white matter connectivity, which are responsible for deteriorating subcortical vascular dementia-related symptoms.

Our clinical results are consistent with previous work, which show disrupted white matter connectivity in patients with the subcortical vascular dementia (SVD) [38], [61], [104]: abnormal frontal-subcortical circuits and disrupted long association fibers. The abnormal frontal-subcortical circuits include brain regions in the prefrontal cortex and subcortical regions such as the anterior cingulate gyrus, the dorsomedial prefrontal cortex, the orbital prefrontal cortex, the inferolateral prefrontal cortex, the middle frontal gyrus, the caudate, the accumbens, the thalamus, and the pallidum. Also, widespread vascular problems may affect the long association fibers including the fiber bundles from the anterior parts to the posterior parts of the brain, such as cingulum, superior longitudinal fasciculus and fronto-occipital fasciculus. On one hand, we observed heavily decreased white matter connectivity in the frontal lobes, especially the prefrontal cortex. In svMCI, the identified sub-network included connections between the prefrontal regions and subcortical regions: the both superior frontal gyri (SFGmed.L, SFGmed.R, and SFGdor.R), the orbital part of the left middle frontal gyrus (ORBmid.L), the both inferior frontal gyri (IFGtri.L and IFGoperc.R), the left anterior cingulate gyrus (ACG.L), the left pallidum (PAL.L), the left putamen (PUT.L), the left amygdala (AMYG.L), and the left thalamus (THA.L). In SVaD, the identified sub-network includes the both superior frontal gyri (SFGdor.L, SFRdor.R and ORBsupmed.R), the both middle frontal gyri (MFG.L,MFG.R, ORBmid.R), the inferior frontal gyrus (IFGtri.L, IFGtri.R, IFGoperc.L and ORBinf.L, ORBinf.R), the left and right putamen (PUT.L and PUT.R), and, the left and right pallidum (PAL.L and PAL.R). These regions are all related to the brain regions in the frontal-subcortical circuits. Because the identified connections were all negatively correlated with the disease severity scores, our results showed the disrupted fronto-subcortical circuits. On the other hand, the identified sub-networks also included the anterior-to-posterior connections, those are correlated with the disease severity (Marked with ‡ in Table 2 and 3). The implication of this result could explain the disruption of long association fibers in patients with SVD [38], [61].

It is interesting to observe that the disease-related network connections identified for each group were not similar to each other. Only three connections were overlapped among about forty connections (38 connections in svMCI and 45 connections in SVaD). The overlapped connections included connections between the left anterior cingulate cortex (ACG.L) and the left precuneus (PCUN.L), between the left pallidum (PAL.L) and the left precuneus (PCUN.L), and between the left middle temporal gyrus (MTG.L) and the left inferior parietal lobule (IPL.L). The first two connections were anterior-to posterior long associate connections. Considering that svMCI is an earlier stage of SVaD, this exclusiveness in the identified sub-networks has some implications. On one hand, the result implies that the specific sub-network of the white matter connectivity disrupted in subjects with svMCI is not much correlated with the disease severity once they become clinically impaired. This could be explained from the fact that the identified set of connections in the svMCI group was already disrupted too much at the early stage. On the other hand, some brain connectivity is significantly disrupted as the disease develops further, while those connections are not that altered at the stage of mild cognitive impairment. Indeed, it is well-known that brain regions that might be associated with the pathology of the Alzheimer’s disease (AD) gradually expand as the disease develops from mild cognitive impairment to severe AD. Our finding therefore implies that such phenomenon in AD is also valid in SVD: the disruption of the white matter connectivity occurs as different locations along the course of the disease severity. The existence of such exclusive sub-networks that are affected by svMCI and SVaD is worthy to be further investigated in future.

**Figure 6:**
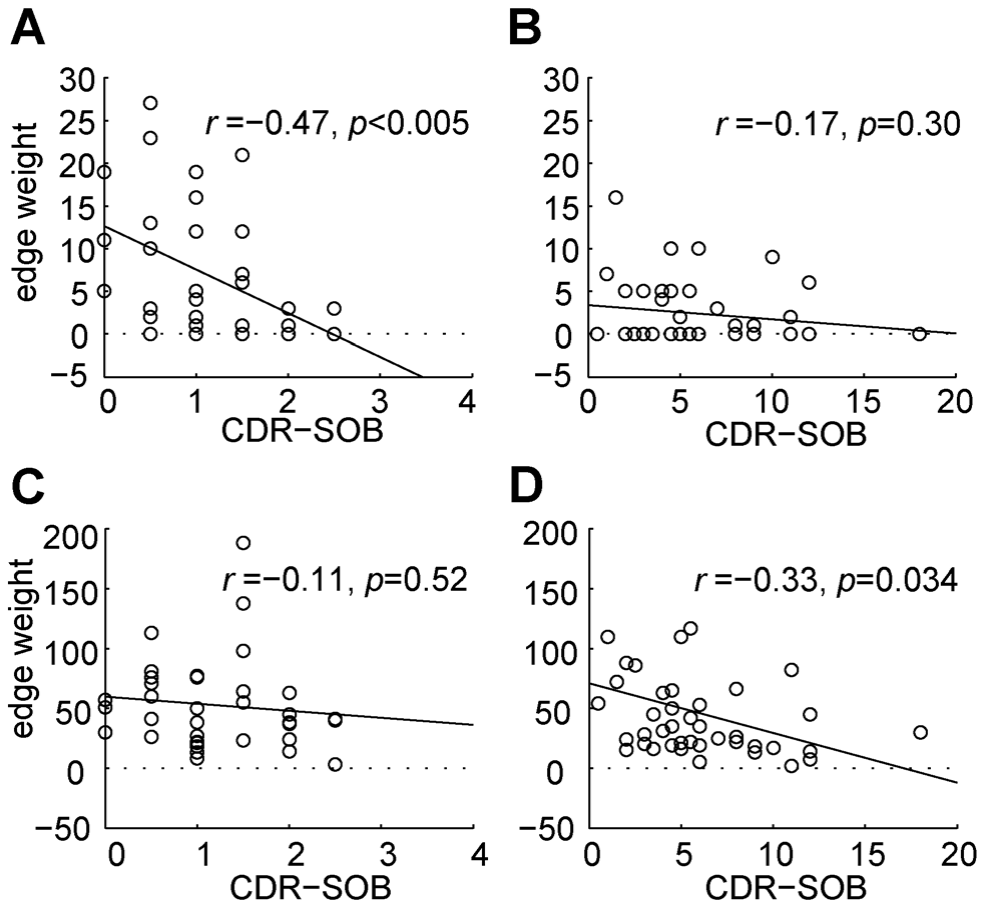
Edge weights over the disease severity score for two representative edges of the identified sub-networks in patients with svMCI and SVaD: the edge between the medial surface of left superior frontal gyrus (SFGmed.L) and the left median cingulate and paracingulate gyri (DCG.L) in patients with svMCI (A) and SVaD (B); and the edge between the orbital part of left inferior frontal gyrus (ORBinf.L) and left insula (INS.L) in patients with svMCI (C) and SVaD (D). A circle represents each subject, dotted horizontal lines represent zero edge weights, and solid lines represent linearly regressed lines. The noted correlation coefficient and *p*-values were calculated using Pearson correlation without any covariates. The former edge (A&B) belonged to the identified sub-network in patients with svMCI, and thus had a strong correlation coefficient in patients with svMCI; however, in patients with SVaD, about half of the subjects had zero or very low weights (20 over 45), resulting in a low correlation coefficient. The latter edge (C&D) belonged to the identified sub-network in patients with SVaD, and thus had a strong correlation coefficient in patients with SVaD; however, in patients with svMCI it is not the case.

Further, we observed different patterns in the scatter plots of the edge weights with regard to the disease severity between the two groups: svMCI and SVaD. For simplicity, we discuss about this observation using two representative network connections: the connection between the medial surface of the left superior frontal gyrus (SFGmed.L) and the left median cingulate and paracingulate gyri (DCG.L) from the identified sub-network in patients with svMCI and the connection between the orbital part of the left inferior frontal gyrus (ORBinf.L) and the left insula (INS.L) for SVaD (Figure 6). We selected those connections because they are dominantly correlated to the disease severity score. Those connections did not have strong correlation with age (Spearman, |*r*| < 0.18) and gender (Spearman, |*r*| < 0.26), and thus their simple correlation coefficients with the sole disease severity without covariates may considerably capture their behavior over the disease severity with the similar large magnitudes (the former edge in svMCI: *r* =  –0.54; the latter edge in SVaD: *r* =  –0.41). In Figure 6, we showed their linear regression lines to delineate the trend of changes. To avoid confusion, we showed their Pearson correlation coefficients, whose trends were similar to the Spearman partial correlation coefficients. The former connection decreases drastically in patients with svMCI, showing strong correlation; however, in patients with SVaD, about half of subjects had zero or very low edge weights (20 among 41), resulting low correlation (Figure 6 A&B). The latter connection sustained its edge weight with the level of around fifty in patients with svMCI and decreased drastically in patients with SVaD, showing strong correlation only in patients with SVaD (Figure 6 C&D).

Strengths of the present study include the comprehensive scan protocol, including high quality clinical diffusion MRI data. Specifically, our data set includes only patients with pure subcortical vascular cognitive impairment, excluding all subjects who showed positive Pittsburgh compound-B (PiB) in PET scans [73]. It is well-known that in the early stage of cognitive impairment, it is often too hard to distinguish other forms of cognitive impairment such as Alzheimer’s disease (AD) from the subcortical vascular cognitive impairment only with the symptoms. Employing PET scans with Pittsburgh compound-B, we excluded other forms of cognitive impairment whose cortices were deteriorated rather white matter connectivity.

### Methodological Limitations

Our method exploits a partial correlation coefficient as a primary statistics. Specifically, in our clinical application, the Spearman correlation coefficient was used to model the relationship between the edge weight and the disease severity. This model, however, is limited to capture complex relationship, such as a quadratic (parabolic) function. In order to resolve this issue, one may employ the general linear model. However, as in our clinical application, if one can expect a monotonic mapping between an edge attribute and a behavioral measure, then the simple partial correlation coefficient might be sufficient.

Similar to the network-based statistics (NBS), the cluster-based approach for multiple comparison correction requires an arbitrary selection of an initial threshold. In our experiments, we searched a range of values for threshold, and selected one with stable results. In order to avoid this empirical selection of initial thresholds, one may employ the threshold-free cluster estimation method proposed by Smith and Nichols [59].

## Conclusion

In this paper, we proposed a cluster-based method for identifying sub-networks that are significantly correlated with behavioral measures. Our method exploits the partial correlation coefficient for modeling the relationship between network connections and a specific behavioral measure of interest, while taking other measures as covariates. We validated the efficacy of our method using clinical data involving patients with subcortical vascular dementia: the results showed that our method is statistically valid but more powerful than Bonferroni correction, FDR procedure, and extreme statistics. We expect wide applications of the proposed method because it does not depend on methods of network construction. The proposed method can be employed not only for the structural/functional brain connectivity but also for edge-specific network measures such as edge betweenness centrality [105], [106]. Also, besides severity scores of a certain disease, any behavioral measure can be correlated with the brain network. For examples, it is feasible to identify sub-networks correlated with age/intelligence in healthy subjects or with hallucination in patients with schizophrenia using a sub-score of PANSS scale [107].

## Supporting Information

Figure S1 **Mapping between a correlation coefficient and** ***t*** **-statistics for varying number of subjects.**
(TIF)

Table S1 **Abbreviated name of brain regions in AAL90.**
(DOCX)
