# Supplementary material for: Cluster-Based Statistics for Brain Connectivity in Correlation with Behavioral Measures
Source: PLoS One. 2013 Aug 19;8(8):e72332. doi: 10.1371/journal.pone.0072332 (PMC3747142; doi:10.1371/journal.pone.0072332)
Supplement: Table S1 — Abbreviated name of brain regions in AAL90. (DOCX) [file pone.0072332.s002.docx]

**Table S1. Abbreviated name of brain regions in AAL90.**

| Brain Region Label | Abbreviation | Brain Region Label | Abbreviation |
| --- | --- | --- | --- |
| Precentral gyrus | PreCG | Lingual gyrus | LING |
| Superior frontal gyrus, dorsolateral | SFGdor | Superior occipital gyrus | SOG |
| Superior frontal gyrus, orbital part | ORBsup | Middle occipital gyrus | MOG |
| Middle frontal gyrus | MFG | Inferior occipital gyrus | IOG |
| Middle frontal gyrus, orbital part | ORBmid | Fusiform gyrus | FFG |
| Inferior frontal gyrus, opercular part | IFGoperc | Postcentral gyrus | PoCG |
| Inferior frontal gyrus, triangular part | IFGtri | Superior parietal gyrus | SPG |
| Inferior frontal gyrus, orbital part | ORBinf | Inferior parietal, but supramarginal and angular gyri | IPL |
| Rolandic operculum | ROL | Supramarginal gyrus | SMG |
| Supplementary motor area | SMA | Angular gyrus | ANG |
| Olfactory cortex | OLF | Precuneus | PCUN |
| Superior frontal gyrus, medial | SFGmed | Paracentral lobule | PCL |
| Superior frontal gyrus, medial orbital | ORBsupmed | Caudate nucleus | CAU |
| Gyrus rectus | REC | Lenticular nucleus, putamen | PUT |
| Insula | INS | Lenticular nucleus, pallidum | PAL |
| Anterior cingulate and paracingulate gyri | ACG | Thalamus | THA |
| Median cingulate and paracingulate gyri | DCG | Heschl gyrus | HES |
| Posterior cingulate gyrus | PCG | Superior temporal gyrus | STG |
| Hippocampus | HIP | Temporal pole: superior temporal gyrus | TPOsup |
| Parahippocampal gyrus | PHG | Middle temporal gyrus | MTG |
| Amygdala | AMYG | Temporal pole: middle temporal gyrus | TPOmid |
| Calcarine fissure and surrounding cortex | CAL | Inferior temporal gyrus | ITG |
| Cuneus | CUN |  |  |
